# Outcomes of early rhythm control vs. rate control strategies for atrial fibrillation in late elderly patients: a systematic review and meta-analysis

**DOI:** 10.3389/fcvm.2025.1700921

**Published:** 2025-12-09

**Authors:** Soban Ahmad, Muhammad Junaid Ahsan, Kathryn M. Kim, Mohammad Hamza Bin Abdul Malik, Sundus Ikram, Michael H. Kim

**Affiliations:** 1 Division of Cardiovascular Medicine, University of Nebraska Medical Center, Omaha, NE, United States; 2 Department of Cardiovascular Medicine, Baylor University Medical Center, Dallas, TX, United States; 3 Department of Internal Medicine, University of Nebraska Medical Center, Omaha, NE, United States; 4 Department of Internal Medicine, Nassau University Medical Center, East Meadow, NY, United States; 5 Department of Internal Medicine, East Carolina University, Greenville, NC, United States; 6 Department of Medicine, Creighton University School of Medicine and CHI Health, Omaha, NE, United States

**Keywords:** atrial fibrillation, rhythm control, rate control, stroke, mortality

## Abstract

Atrial fibrillation (AF) is highly prevalent in late elderly patients, yet optimal management remains uncertain. While early rhythm control has shown benefits in younger populations, evidence in patients over 75 years is limited, and rate control remains the main treatment strategy. We performed a systematic review and meta-analysis comparing early rhythm vs. rate control in elderly AF patients. Seven retrospective studies with a total of 96,699 patients (27,771 rhythm control; 68,928 rate control) were analyzed. The mean ages were 78.1 and 79.5 years in the rhythm and rate control groups, respectively. Rhythm control was associated with a lower risk of stroke (RR 0.82, *p* = 0.02) but a higher risk of permanent pacemaker implantation (RR 2.54, *p* = 0.04). All-cause mortality and cardiovascular mortality were similar between the two groups. Our findings suggest that while early rhythm control may reduce stroke risk in the late elderly, it carries higher device-related risks without a mortality benefit. Treatment goals should be individualized and guided by patient comorbidities, preferences, and overall frailty.

## Introduction

Older age is a major non-modifiable risk factor for atrial fibrillation (AF). AF prevalence increases from ∼3% in those aged 65 years or older to ∼14% in those aged 80 years or older. Management of AF in the elderly presents significant challenges due to the complex interplay of comorbidities, elevated risks of stroke and bleeding, drug-drug interactions due to polypharmacy, and potential adverse effects of anti-arrhythmic drugs (AADs). Contemporary data with either early rhythm control have shown improved outcomes as compared to a rate control strategy. Despite these findings, rate control remains the main treatment strategy in many elderly patients, largely due to limited evidence to support rhythm control, especially in the late elderly over 75 years of age. To address this knowledge gap, a systematic review and meta-analysis was performed to evaluate outcomes of an early rhythm vs. rate control strategy in elderly AF patients.

## Methodology

We searched MEDLINE, EMBASE, Scopus, Cochrane CENTRAL, conference abstracts, and ClinicalTrials.gov for studies including AF patients aged >65 years, through March 19, 2024. Only English-language studies were considered. The primary outcome was all-cause mortality. Pooled risk ratios (RRs) with their 95% confidence intervals (CIs) were calculated using RevMan 5.4 software.

## Results

**Figure 1:**
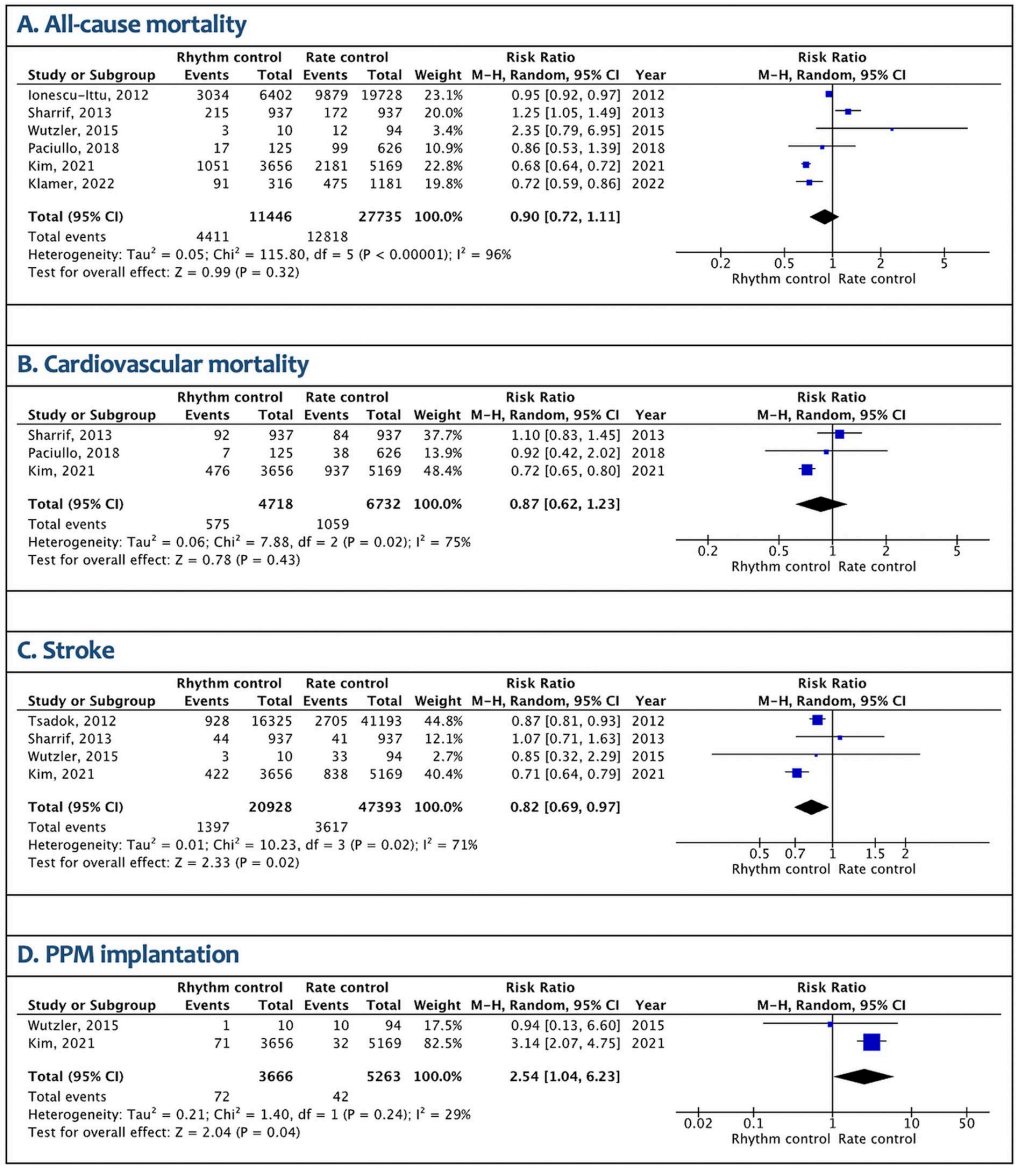
Outcomes of early rhythm control vs. rate control strategies for atrial fibrillation in late elderly patients. **(A)** All-cause mortality. **(B)** Cardiovascular mortality. **(C)** Stroke. **(D)** PPM implantation.

Seven retrospective studies comprising a total of 96,699 patients (rhythm control: 27,771 and rate control: 68,928) were included in this analysis (1–7). Included studies defined early rhythm control as initiation of AAD or ablation within 30 days to 1 year after initial AF diagnosis. The mean age was 78.1 years in the rhythm control group and 79.5 years in the rate control group, with females comprising 52% and 44% of the rhythm and rate control cohorts, respectively. Rhythm control was associated with reduced stroke risk (RR 0.82, 95% CI 0.69–0.97; *p* = 0.02; *I*^2^ = 71%) but higher pacemaker implantation risk (RR 2.54, 95% CI 1.04–6.23; *p* = 0.04; I²=29%). Mortality outcomes showed no significant difference, though heterogeneity was extreme (all-cause mortality: RR 0.90, *I*^2^ = 96%; cardiovascular mortality: RR 0.87, *I*^2^ = 75%) (Figures 1A–D).

## Discussion

This study compared the effectiveness of rhythm control vs. rate control in late elderly AF patients with a mean age of 78.1 years and 79.5 years in the rhythm control and rate control groups, respectively. The analysis showed that despite a similar rate of anticoagulation use (80.1% vs. 79.9%) between patients treated with rhythm and rate control strategies, stroke risk was much lower with rhythm control. This finding is supported by the Kaiser Permanente Real-World Heart Monitoring Strategy Evaluation, Treatment Patterns, and Health Metrics in Atrial Fibrillation (KP-RHYTHM) study, which showed that a greater burden of atrial fibrillation is associated with a higher risk of thromboembolism, regardless of known stroke risk factors. Our results are consistent with the landmark Early Rhythm-Control Therapy in Patients with Atrial Fibrillation (EAST-AFNET 4) trial that showed a lower risk of stroke with early rhythm control. Furthermore, two recent meta-analyses have reported that catheter ablation is associated with a lower risk of stroke when compared to medical therapy for AF (8, 9). Notably, patients included in both meta-analyses were younger and had lower female representation compared to our study.

Studies included in the meta-analysis primarily achieved rhythm control using class I and III AADs. Notably, only one study utilized catheter ablation (CA) as a rhythm control strategy (6). AADs use may impair sinoatrial node function due to their effect on a broad spectrum of ion channels and beta-adrenergic receptors. Additionally, the presence of underlying sinus node dysfunction (SND) in frail elderly patients may make them more susceptible to the side effects of AADs. Okumus et al. reported that compared to AAD use, CA was associated with a significantly lower risk of PPM (hazard ratio 0.58) in patients with AF and underlying SND (10). AAD use as the primary rhythm control strategy in the included studies may potentially explain the increased risk of PPM implantation in our analysis.

These data showed that the risk of cardiovascular and all-cause mortality may be similar between rhythm and rate control cohorts in late elderly patients. The absence of mortality benefit may reflect the older age and higher comorbidity burden of included patients. This hypothesis is supported by a subgroup analysis from the Registry on Cardiac Rhythm Disorder Assessing the Control of Atrial Fibrillation (RECORD-AF) study, which showed that poor outcomes in AF patients are primarily influenced by the presence of comorbidities, such as congestive heart failure, chronic kidney disease, coronary artery disease and a prior history of stroke, rather than the choice of rate vs. rhythm strategy (11). Additionally, a recent meta-analysis showed similar success rates for AF ablation in elderly (>75 years) patients compared to younger individuals; however, ablation was associated with a higher complication rate (12). Lastly, a high prevalence of AAD use instead of ablation in the rhythm control arm may also explain the lack of mortality benefit.

We acknowledge several limitations of this meta-analysis: Firstly, all included studies were retrospective with heterogeneous definitions of early rhythm control (30 days to 1 year). Rhythm control strategy was mainly AAD-based, with only one study utilizing ablation (6). Lastly, statistical heterogeneity for multiple endpoints was high despite the use of random effects modeling.

## Conclusion

This meta-analysis of retrospective studies shows that early rhythm control is associated with reduced stroke risk in late elderly AF patients but increases the likelihood of pacemaker implantation. Both early rhythm and rate control strategies had similar mortality rates. Given the limitations of this study, we recommend further research to better understand long-term outcomes of early rhythm control in the late elderly population.
